# High school versus graduate entry in a Saudi medical school – is there any difference in academic performance and professionalism lapses?

**DOI:** 10.1186/s12909-016-0834-7

**Published:** 2016-12-19

**Authors:** Ahmed Rumayyan Al Rumayyan, Abdulaziz Ahmed Al Zahrani, Tahir Kamal Hameed

**Affiliations:** 1 Department of Pediatrics, King Abdullah Specialized Children’s Hospital, College of Medicine, King Saud bin Abdulaziz University for Health Sciences, King Abdulaziz Medical City, Ministry of National Guard – Health Affairs, PO Box 3660, Riyadh, 11481 Saudi Arabia; 2 Department of Anesthesiology and Critical Care, King Fahad Specialist Hospital – Dammam, Dammam, Saudi Arabia; 3 Department of Pediatrics, King Abdullah Specialized Children’s Hospital, King Abdulaziz Medical City, Ministry of National Guard – Health Affairs, College of Medicine, King Saud bin Abdulaziz University for Health Sciences, PO Box 22490, Riyadh, 11426 Saudi Arabia

**Keywords:** Medical school, School admission criteria, Graduate entry medicine, Educational measurement, Professionalism

## Abstract

**Background:**

King Saud bin Abdulaziz University for Health Sciences (KSAU-HS) was the first university in the Kingdom of Saudi Arabia offering both high school entry and graduate entry (GE) students into medical school. We compared the academic performance and professionalism lapses of high school entry and GE students who undertook the same curriculum and examinations in the College of Medicine, Riyadh, KSAU-HS.

**Methods:**

Examination scores of 196 high school graduates and 54 GE students over a 4-year period (2010–2014) were used as a measure of academic achievement. For assessment of professionalism lapses, we compared the number of warning letters in both streams of students.

**Results:**

In some pre-clinical courses, high school entry students performed significantly better than GE students. There was no significant difference in academic performance of high school entry and GE students in clinical rotations. GE students had a significantly greater number of warning letters per student as compared to high school entry students.

**Discussion:**

This is the first Saudi study to compare the performance of high school entry and GE students in a medical school. Overall, both streams of students performed equally well with high school entry students performing better than GE students in a few pre-clinical courses. We compared professionalism lapses and found an increase in number of warning letters for GE students. More studies are needed to evaluate if there are differences in other assessments of professionalism between these two streams of students.

## Background

There has been a substantial increase in the number of graduate entry (GE) programs in medical schools over the last decade in the UK, Ireland and Australia. The most recent study comparing high school and GE students was reported from Ireland and found that GE students performed at least as well, or even better than high school entry students in both bioscience knowledge and clinical assessments [1]. In a study from Australia, Reid et al. [2] found that both GE and high school entry medical students performed similarly on clinical assessments. The same study group found that in pre-clinical assessments, GE students had a marginal academic performance advantage [3]. In a large-scale UK study to compare the performance of GE and school-leaver (high school) entry medical students, Shehmar et al. [4] found that high school entry students performed better on clinical examinations midway through training, but there was no significant difference in the final clinical exam. The study also showed no significant difference between the groups in performance of the Phase I (basic sciences knowledge) exam. A study from the University of Nottingham, UK found that GE students outperformed high school entry students in the initial clinical phase, but had a lower performance on most of the subsequent knowledge-based exams [5]. Finally, another study from the UK reported that on average the academic performance of GE medical students was better than high school entry students [6]. Based on this literature, there is no clear advantage of one group over the other in terms of academic performance. To the best of our knowledge, the academic performance of high school entry and GE students has not been studied in the Kingdom of Saudi Arabia or in the Gulf region.

GE into medical school was first introduced in Saudi Arabia at King Saud bin Abdulaziz University of Health Sciences (KSAU-HS) in 2005. Moreover, KSAU-HS was the first specialized university dedicated for health sciences training in the Gulf region. It began as one main branch in Riyadh, and there are now two more branches in Jeddah (Western Region) and Al-Hasa (Eastern Region). When the College of Medicine, Riyadh (COM-R) opened in 2005, admission was only given to GE students; in fact, the first five batches of medical students were only GE students. In 2007, high school entry students were admitted to the COM-R. GE students complete an introductory 4-month curriculum followed by the 4-year medicine course (which includes 2 years of pre-clinical courses structured as systems-based blocks followed by approximately 2 years of clinical rotations). High school entry students complete a 6-year curriculum that includes a 2-year pre-professional programme followed by the 4-year medicine programme. Their 4-year medicine programme is identical to the 4-year programme that GE students take. Thus these two cohorts of students undertake the same curriculum and are subjected to the same assessment exams (written, OSPEs and OSCEs).

While other studies have compared academic performance between high school entry and GE students, we also sought to address any differences in professionalism lapses between these two groups of students. The definition of professionalism and the best practices for its assessment remains complex [7]. Common elements of professionalism include altruism, respect for others, honour, integrity, ethical and moral standards, accountability, excellence, and duty. Assessment of professionalism must be integrated across the medical course using multiple tools [8]. A major assumption in medical education is that professional students become professional physicians [9]. In two landmark studies, unprofessional behaviour in medical school was associated with subsequent disciplinary action against practicing physicians [9, 10]. Our research objectives were to compare the academic performance of high school graduates and GE medical students in the COM-R, and to compare professionalism lapses between these two streams of students.

## Methods

A retrospective quantitative cohort study was carried out to compare academic performance and professionalism lapses of high school entry and GE entry students in the COM-R, KSAU-HS over a 4 year period (2010–2014). The study included 3 cohorts of students in which there were both streams of students (high school entry and GE). These cohorts of students were all males and included students in Batches 7, 8, and 9. Female students were enrolled in the COM-R from Batch 8; however as their numbers were small and only high school entry, their data was not included in the analysis. Academic Affairs in the College of Medicine provided the final grades (given in percentages) for the students. Academic performance was compared for pre-clinical courses (all batches) and clinical rotations (Batches 7 and 8). At the time of the conclusion of the study, Batch 7 students had graduated in 2014, Batch 8 students had not completed all clinical rotations and Batch 9 students were in their first year of clinical rotations. The pre-clinical courses for both streams of students included Foundation studies, Musculoskeletal Sciences, Respiratory Sciences, Hematology, Cardiovascular Sciences, Medical Elective I, Neurosciences Vision and Behavior, Endocrine, Nutrition & Reproductive Health, Urology & Renal, Gastroenterology & Nutrition, and Oncology & Palliative Care. The final grade for these courses (except Medical Elective I) included results from mid-term and final examinations (written) as well as OSPEs and OSCEs. The clinical rotations in which we had data for Batches 7 and 8 included Surgery I, Medicine I, Pediatrics, and Family & Community Medicine. The assessment for these rotations also included written examinations and OSCEs as well as the Mini-Clinical Evaluation Exercise (Mini-CEX). Data supplied to the researchers consisted of final grades classified by stream (high school entry vs. GE) without any names of students. The grades were reported as percentages and they were obtained from gathering examination results of each student in each block.

Student Affairs provided the number of warning letters students received by stream of student. Warning letters are given if students violate the code of ethics (for example: excessive absences, smoking on campus, signing an attendance sheet and then leaving, etc.). Warning letters data was available for both streams of students in Batches 7 and 8.

For academic performance, the mean final percentage for each block or clinical rotation was calculated for each stream of students. Data analysis was performed with SPSS version 22 (IBM Corporation, USA). The independent samples *t*-test compared the means between the two unrelated groups on the same continuous dependent variable. Minitab version 17 was used to perform the test of equality of the Poisson rates (mean) for warning letters. Significant differences were identified at *P* < 0.05. Approval for our study was obtained from the Research Office at King Abdullah International Medical Research Center, Ministry of National Guard - Health Affairs as well as the Dean, COM-R, KSAU-HS.

## Results

High school entry students accounted for 196 (78%) of the sample as compared to 54 (22%) graduate entry (GE) students (Table 1). Virtually all of GE students had Bachelor’s degrees in health science fields including clinical laboratory sciences, pharmacy, respiratory therapy, etc. In the pre-clinical phase, there was no significant difference in the overall percentage grade between high school entry and GE students. However, the percentage grade for high school entry students was significantly higher than GE students in 3 courses (Foundation Studies, Urology & Renal, and Gastroenterology & Nutrition blocks) (Table 2). There was no significant difference in academic performance for four clinical rotations in which there were 118 and 34 high school entry and GE students, respectively (Table 3). Warning letter data was obtained for Batch 7 and 8 students, and 105 students (69%) received at least one warning letter. The average number of warning letters per student was 10.15 for high school entry students and 14.91 for GE students, which was statistically significant (Table 4) (*p* < 0.001).

**Table 1:** Number of high school entry and graduate entry students in each cohort

	Cohort			
	1 (Batch 7)	2 (Batch 8)	3 (Batch 9)	Total
High school entry	42	76	78	196
Graduate entry	18	16	20	54

**Table 2:** Mean percentages and Standard Deviations (SDs) of high school entry and graduate entry students in pre-clinical courses

Course	High school entry students, *N* = 196		Graduate entry students, *N* = 54			
	Mean, %	SD	Mean, %	SD	*p*-value	Cohen’s *d*
Foundation Studies	81.3	7.2	78.2	7.0	*0.01*	0.44
Musculoskeletal Sciences & Substance Abuse	79.0	7.7	77.3	7.3	0.13	0.23
Respiratory Sciences	81.7	7.4	81.5	7.3	0.80	0.03
Hematology	83.9	7.2	84.1	8.6	0.87	0.03
Cardiovascular Sciences	83.0	7.6	81.2	6.6	0.12	0.25
Neurosciences, Vision & Behavior	82.3	8.0	80.4	7.0	0.10	0.25
Endocrine, Nutrition & Reproductive Health	81.9	8.5	79.6	7.7	0.09	0.28
Urology & Renal	85.2	5.7	82.7	7.0	*0.02*	0.39
Gastroenterology & Nutrition	82.6	7.0	80.4	6.3	*0.04*	0.33
Oncology & Palliative Care	85.8	6.2	85.2	5.7	0.48	0.10
Overall percentage in pre-clinical courses	82.7		81.1		0.12

**Table 3:** Mean percentages and Standard Deviations (SDs) of high school entry and graduate entry students in clinical rotations

Clinical rotation	High school entry students, *N* = 118		Graduate entry students, *N* = 34			
	Mean, %	SD	Mean, %	SD	*p*-value	Cohen’s *d*
Medicine I	85.1	5.8	84.9	5.9	0.81	0.03
Surgery I	81.5	6.3	80.5	5.3	0.37	0.17
Pediatrics	85.4	4.7	84.0	5.5	0.15	0.27
Family & Community Medicine	83.3	4.9	81.6	4.4	0.06	0.37
Overall percentage in clinical rotations	83.8		82.8		0.46

**Table 4:** Warning letters for high school entry and graduate entry students

	High school entry students	Graduate entry students	*p*-value
Average number of warning letters per student	10.15	14.91	<0.001

## Discussion

Our results show that high school entry students performed significantly better than graduate entry (GE) on a few pre-clinical courses, with no significant difference in academic performance between the two groups in clinical rotations. Our study also compared some professionalism lapses between these two groups of students and we found a significant increase in the number of warning letters per student for the GE stream.

Our finding of essentially equal academic performance between high school and GE students is supported by other recent studies conducted in UK, Ireland, and Australia [1–6]. While there are now several studies comparing academic performance of high school entry and GE students into medical school, our study was the first to look at some professionalism lapses between the two streams of students. We found that GE students received a significantly higher number of warning letters as compared to high school entry students. This finding is somewhat surprising as GE students are considered to be more mature than non-graduate medical students [11]. In addition, one study focusing on the attitudes towards professionalism in these two groups of students found that GE students ascribed greater importance to various aspects of professionalism across personal characteristics, interaction with patients and social responsibility categories [12]. In our medical school, GE students were the minority and they may feel more “alienated” in the medicine programme [13]. Furthermore, though GE students are expected to be more mature, there may be other factors like family responsibilities, stress and burnout that have contributed to our finding of more professionalism lapses observed in this group. Further research is needed to assess these factors.

There are several strengths of our study. First, this was the first study in Gulf region to compare academic performance of high school and GE students. We used direct comparison of performance for both groups of students who undertook the same curriculum and same examinations. Furthermore, while other studies compared academic performance, our study was the first to evaluate differences in professionalism lapses between these two groups of students. There are limitations of this study. The main limitation of this study is the small number of high school (196) and GE (54) students limiting the generalizability of our results. In addition, only male students were included in the analysis as female students started from Batch 8 but there were only high school entry students in that cohort. Finally, the assessment of professionalism is more complex than simply the number of warning letters per student, and other professionalism measures are needed to compare professionalism between the two groups.

## Conclusions

High school entry and GE medical students perform equally well on clinical rotations, with high school entry students performing significantly better on some pre-clinical courses. There was a significant difference in number of warning letters per student between the two groups, with GE students receiving a higher number as compared to high school entry students. Further studies are needed to evaluate whether differences exist between high school and GE students on other assessments of academic performance (including during residency training and beyond) and on other assessments of professionalism.

## Abbreviations

COM-R: College of Medicine, Riyadh
GE: Graduate entry
KSAU-HS: King Saud bin Abdulaziz University for Health Sciences
OSCE: Objective structured clinical examination
OSPE: Objective structured practical examination

## References

[1] Byrne AT, Arnett R, Farrell T, Sreenan S (2014). Comparison of performance in a four year graduate entry medical programme and a tradiational five/six year programme. BMC Med Educ.

[2] Reid KJ, Dodds AE, McColl GJ (2012). Clinical assessment performance of graduate- and undergraduate-entry medical students. Med Teach.

[3] Dodds AE, Reid KJ, Conn JJ, Elliott SL, McColl GJ (2010). Comparing the academic performance of graduate- and undergraduate-entry medical students. Med Educ.

[4] Shehmar M, Haldane T, Price-Forbes A, Macdougall C, Fraser I, Paterson S, Peile E (2010). Comparing the performance of graduate-entry and school-leaver medical students. Med Educ.

[5] Manning G, Garrud P (2009). Comparative attainment of 5-year undergraduate and 4-year graduate entry medical students moving into foundation training. BMC Med Educ.

[6] Calvert MJ, Ross NM, Freemantle N, Xu Y, Zvauya R, Parle JV (2009). Examination performance of graduate entry medical students compared with mainstream students. J R Soc Med.

[7] Hodges BD, Ginsburg S, Cruess R, Cruess S, Delport R, Hafferty F, Ho MJ, Holmboe E, Holtman M, Ohbu S, Rees C, Ten Cate O, Tsugawa Y, Van Mook W, Wass V, Wilkinson T, Wade W (2011). Assessment of professionalism: Recommendations from the Ottawa 2010 Conference. Med Teach.

[8] O’Sullivan H, Van Mook W, Fewtrell R, Wass V (2012). Integrating professionalism into the curriculum: AMEE Guide No. 61.. Med Teach.

[9] Papadakis MA, Teherani A, Banach MA, Knettler TR, Rattner SL, Stern DT, Veloski JJ, Hodgson CS (2005). Disciplinary Action by Medical Boards and Prior Behavior in Medical School. NEJM.

[10] Papadakis MA, Hodgson CS, Teherani A, Kohatsu ND (2004). Unprofessional Behavior in Medical School Is Associated with Subsequent Disciplinary Action by a State Medical Board. Acad Med.

[11] McCrorie P (2002). Graduate students are more challenging, demanding, and questioning. BMJ.

[12] O’Flynn S, Power S, Horgan M, O’Tuathaigh CMP (2014). Attitudes towards Professionalism in Graduate and Non-graduate Entrants to Medical School. Educ Health.

[13] Sefton AJ (2004). Graduate entry to medical school. Med Educ.

